# In Vitro Robotic Chewing Studies of Food Texture Changes During Mastication

**DOI:** 10.1111/jtxs.70037

**Published:** 2025-08-26

**Authors:** Xudong Wang, Bangxiang Chen, Jaspreet Dhupia, Macro Morgenstern, Weiliang Xu

**Affiliations:** ^1^ Department of Mechanical & Mechatronics Engineering The University of Auckland Auckland New Zealand; ^2^ School of Advanced Technology Xi'an Jiaotong‐Liverpool University Suzhou China; ^3^ New Zealand Institute for Plant and Food Research Lincoln New Zealand

**Keywords:** chewing behaviors, food texture change, mastication, robotic chewing

## Abstract

The aim of this study was to investigate food texture changes during mastication using a robotic chewing system. Roasted peanuts and white bread were used as representative food samples to explore how different chewing behaviors affect textural transformations. Initially, the number of cycles required to chew each food sample for swallowing was recorded through in vivo experiments. Subsequently, various molar chewing trajectories, occlusal forces, and artificial saliva flow rates were applied in the robotic chewing system to simulate a range of chewing behaviors. Each food type was chewed by the robot for 0%, 25%, 50%, 75%, and 100% of the total determined number of chewing cycles. Nine food texture variables were measured using texture profile analysis (TPA). Principal component analysis (PCA) and partial least squares regression (PLSR) were employed to assess the correlations between chewing behaviors and food texture changes. Results showed that for roasted peanuts, hardness, adhesive force, and cohesiveness had strong correlations with chewing cycles, while for white bread, these relationships were less pronounced. The mechanisms underlying the texture changes were analyzed and explained. For roasted peanuts, texture changes were primarily governed by chewing stages, with an average hardness reduction of 81.5% over the chewing process. Springiness and its index were mainly influenced by saliva secretion rate. Conversely, white bread initially exhibited increased hardness due to compression, followed by gradual softening. Its adhesive force was chiefly impacted by saliva secretion, while cohesiveness was more strongly affected by chewing trajectory. These findings should be interpreted cautiously, as the limited and homogeneous participant sample restricts their broader applicability.

## Graphical abstract

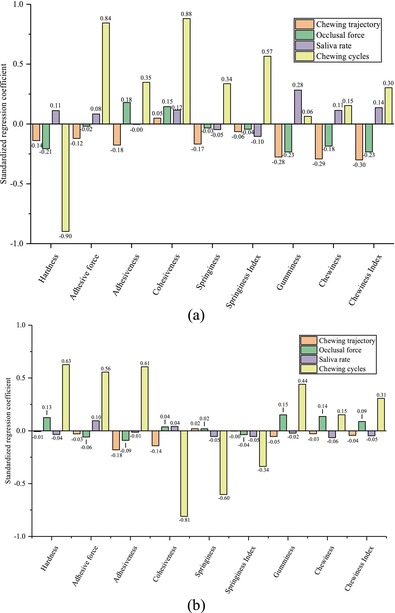

## 1 Introduction

Food oral processing (FOP) is a multifaceted biomechanical and biochemical process that encompasses mastication, salivation, bolus formation, and swallowing (Wang and Chen 2017; He et al. 2022). During this process, solid foods are broken down into smaller fragments and thoroughly mixed with saliva, forming a semi‐solid, cohesive, and deformable bolus that can be safely swallowed. The sensory and functional manifestation of the structural, mechanical, and surface properties of foods, detected in the mouth, is referred to as food texture (Chen and Rosenthal 2015); changes during FOP. The dynamic changes in food texture and its sensory perception throughout FOP play a crucial role in flavor release, nutrient bioavailability, and ease of swallowing, all of which are essential for effective digestion and optimal nutrient absorption. Numerous in vivo studies have been conducted to investigate the impact of food's mechanical properties and sensory attributes perceived during mastication on bolus texture (Pu et al. 2021; Puerta et al. 2020; Almotairy et al. 2021). However, the structural transformations that food undergoes in the oral cavity—where it is compressed, sheared, and mixed with saliva—are highly complex and difficult to replicate or quantify. Traditional methods of studying food breakdown typically rely on either subjective sensory evaluations or isolated mechanical testing (Wada et al. 2017), both of which are limited by substantial individual variability and constrained experimental conditions (Wang and Chen 2017).

In response to these challenges, in vitro methods, particularly robotic mastication simulations, have emerged as valuable tools for systematically studying FOP by replicating human chewing behavior in a controlled and repeatable manner. For instance, the AM2 system, featuring a stationary maxillary disk and a moving mandibular disk housed within a cylindrical cavity, simulates the chewing process through the translation and rotation of the mandibular disk, producing a bolus with properties comparable to those generated by human mastication (Woda et al. 2010; Mishellany‐Dutour et al. 2011). Similarly, the Model Mouth (Salles et al. 2007; Kristiawan et al. 2021) builds upon the AM2 design by incorporating an actuated, cone‐shaped tongue, ring‐shaped molar teeth, and a gas sampling mechanism for monitoring and analyzing volatile organic compounds (VOCs). A linkage‐based chewing robot (Chen et al. 2022) was developed to trace a planned variable molar trajectory while chewing peanuts. The particle size distribution (PSD) and particle median size diameter were studied and compared with in vivo results. An artificial model mouth (Pu et al. 2021) was developed and applied to study the microstructural and chemical composition properties of white bread on food texture changes during oral processing. A novel bio‐inspired oral mastication simulator (iBOMS‐III) (Xu et al. 2024) was developed to generate a bolus of cooked rice and roasted peanuts; the PSDs and the mechanical properties of the bolus were studied and compared with in vivo results to validate its effectiveness.

Additionally, chewing robots designed with human anatomical features have been employed to evaluate food texture and investigate the biomechanics of mastication (Alemzadeh et al. 2021; Peyron and Woda 2016; Mostashiri et al. 2020). For example, a chewing robot with linear actuators and load cells was developed to chew cookies and jellies while measuring the force distribution on teeth (Lee et al. 2018). A bionic chewing robot based on the six‐axis parallel mechanism was developed, and dental wax and peanuts were applied as sample foods to study the particle sizes and mixing effect index (MEI) after chewing (Zhou and Yu 2022). A common feature shared by these robotic systems is their comprehensive modeling of the human oral cavity, including the maxilla, mandible, gums, and complete dental structure, which enhances their ability to closely mimic human mastication dynamics.

The development of existing chewing robots and mastication simulators generally follows two primary technical approaches: mimicking human biomechanics and producing a food bolus. Each approach involves certain compromises. The former category includes robots designed to replicate the biomechanical characteristics of human mastication (Cheng et al. 2016; Wen et al. 2019), while the latter consists of robots aimed at generating a food bolus with properties suitable for subsequent analysis. In the latter approach, food is fragmented into smaller particles through compressive and shear forces generated by the vertical (Xu et al. 2024) movements of the translational teeth or rotational movements of the circular lower jaw (Morell et al. 2014; Peyron et al. 2019) and is thoroughly mixed with artificial saliva to form a cohesive bolus. However, the real chewing motion is greatly simplified; its effect on food texture change and bolus formation is also ignored.

A multifunctional mastication simulator has been developed to investigate various parameters of the mastication process, including food breakdown, chewing force, pH value, conductivity, and aroma release (Zhang et al. 2023). Despite these advancements, the occlusal force in robotic chewing remains difficult to adjust to reflect the diverse chewing conditions across different populations. Sample collection plates are applied to confine food particles within the occlusion area of the upper and lower molars. However, they also restrict the horizontal movement of the upper molar to only 0–5 mm, thus limiting the ability to reproduce lateral shearing, an important component of human mastication.

Existing research has validated the feasibility of chewing robots in replicating the human mastication process by conducting mechanical simulations using single or multiple types of food. Although prior robotic chewing studies explored texture breakdown using simplified systems, limited work has systematically investigated how multiple masticatory parameters affect texture evaluation in a biomimetic and systematic way. A systematic investigation into the mechanism of texture evolution under the combined influence of multiple factors remains insufficient and requires further in‐depth study. Therefore, in this paper, a three‐chamber chewing robot developed in our lab (Wang et al. 2025) was applied to study the effects of different oral conditions on the food texture changes during the masticatory process. This one degree‐of‐freedom (DoF) chewing system allows high‐throughput testing, with cam‐based trajectory modulation to simulate realistic vertical and lateral molar motions. The occlusal force can also be set up via the Remote Centre Compliance (RCC) mechanisms applied to the chewing robot. This study examines four independent variables: chewing trajectory, occlusal force, saliva secretion rate, and food type. The first three variables, which are chewing trajectory, occlusal force, and saliva rate, are each investigated at three distinct levels. Food type consists of two categories: roasted peanuts and white bread. These variables are systematically combined to simulate various chewing behaviors and to evaluate their effects on food texture changes during robotic chewing.

First, the number of chewing cycles required to chew the foods until they were ready to be swallowed was recorded through in vivo experiments. Afterwards, in vitro experiments were conducted using molars operating at a constant speed of approximately 1 s per chewing cycle. The molar trajectories, occlusal force profile, and flow rates with three levels were combined to simulate different chewing behaviors. The food samples were chewed for 0%, 25%, 50%, 75%, and 100% of the number of chews, and the food textures were measured and compared with the in vivo results. Partial least squares regression (PLSR) was applied to analyze the correlation between the chewing behaviors and food texture changes during chewing. Finally, the mechanisms of food texture changes were analyzed.

This study simulates nine distinct chewing behaviors via orthogonal design, integrating biomimetic variation in chewing force, chewing trajectory, and saliva secretion, to investigate how multiple masticatory parameters affect texture evolution in a biomimetic way. The chewing parameters were varied in a parametric manner under controlled experimental conditions to evaluate their individual and combined effects on bolus properties. It provides insights into how food texture changes in response to different chewing behaviors during mastication. The dominant factors affecting the changes in food textures during the masticatory process were analyzed. It can also be an alternative technique for in vitro evaluation of masticatory performance within different groups of people.

## 2 Materials and Methods

### 2.1 Food Samples and Artificial Saliva

Tip Top White Toast Bread (Tip Top Bakery Co. Ltd., Auckland, New Zealand) and roasted peanuts (Griffin's Snacks, Auckland, New Zealand), purchased from Woolworths supermarket (Auckland, New Zealand), were used as sample foods in this study.

The artificial saliva used in this study was Biotene Dry Mouth Relief Mouthwash, an oral rinse that has previously been used as a saliva substitute to study its effect on the mineral content of demineralized and sound dental enamel (Kielbassa et al. 2001) or the tribological behavior of dental implants (Alemanno et al. 2020) primarily for its ease of standardization and prior precedent in mastication‐related research. It was produced by Haleon plc, a British multinational consumer healthcare company, and was purchased from Chemist Warehouse (Auckland, New Zealand). It contains Water, Glycerin, Xylitol, Sorbitol, Propylene Glycol, Poloxamer 407, Sodium Benzoate, Hydroxyethylcellulose, Methylparaben, Propylparaben, Flavor, Sodium Phosphate, Disodium Phosphate. It cannot fully replicate the enzymatic and rheological properties of human saliva and represents only an approximate proxy; however, this product claims to contain a mouth‐moisturizing system to provide smooth relief and help maintain a healthy oral environment, which is similar to the functions of human saliva. This artificial saliva was stored at 4°C prior to the experiments.

### 2.2 Chewing Cycles Determination

The number of chewing cycles required for robotic chewing was determined through in vivo chewing experiments. Two subjects (Both male, 29 years old) were required to chew white bread and peanuts with molars on one side. Ethical approval was obtained from University of Auckland (Ref: UAHPEC26909) before the in vivo study. The number of chews required to chew the food until they were ready to be swallowed was recorded, and each chewing experiment was repeated five times. For peanuts chewing, two kernels of peanuts were chewed every time. For white bread chewing, the bread slices were cut into food samples measuring 3.0 cm in length, 3.0 cm in width, and 1.0 cm in thickness. Results are shown in Table 1. The average number of chews for peanuts for two subjects was 16.8 and 16.0, respectively. Therefore, 16 was selected as the number of chews required for the robotic chewing of peanuts, which is similar to the results published in (McKiernan and Mattes 2010). The average values of bread chewing were 11.6 and 11.4, respectively, therefore, 12 was selected as the number of chews required for robotic chewing of white bread, which is slightly fewer than the 17 chewing cycles reported in the existing literature (Gao and Zhou 2021).

**TABLE 1:** Chewing cycles of subjects when chewing peanuts and bread.

	Subject 1					Subject 2				
	Test 1	Test 2	Test 3	Test 4	Test 5	Test 1	Test 2	Test 3	Test 4	Test 5
Peanuts	18	17	19	14	16	16	14	16	17	17
Bread	13	12	12	11	10	10	11	12	12	12

### 2.3 The In Vitro Chewing Robot

**FIGURE 1:**
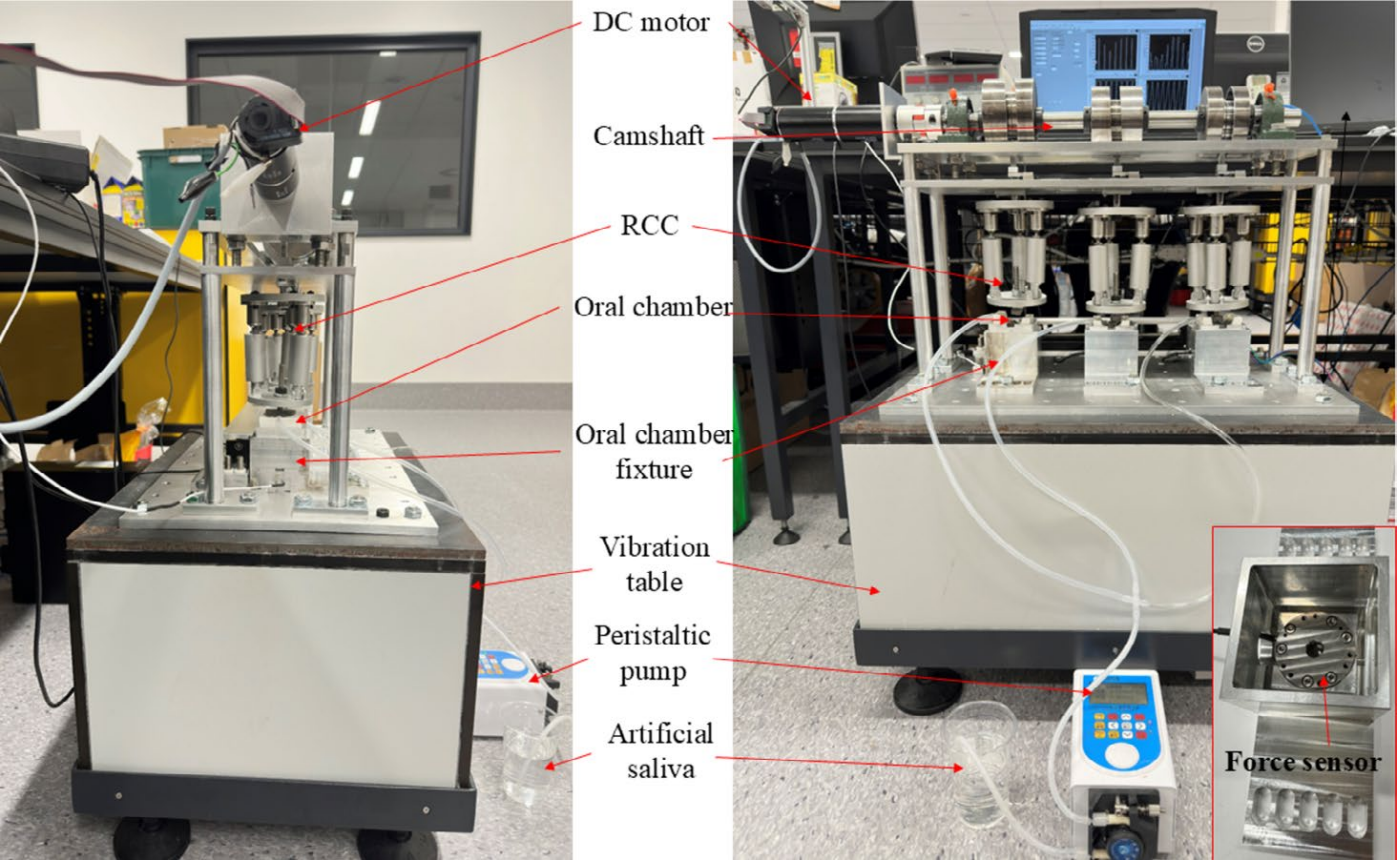
The chewing robot used in the study (Wang et al. 2025).

The chewing robot developed in our lab (Wang et al. 2025), which can simulate the molar's grinding and shearing of foods, was used in this study. This chewing robot shown in Figure 1 has three oral chambers, and the molars are connected to the followers through RCC mechanisms following the chewing movements in a 2D plane guided by the cams. Each chamber follows a unique cam‐guided trajectory generated by specifically designed eccentric cams, enabling the simulation of vertical chewing motion with three levels of lateral chewing motions (0, 3.3, and 10 mm). The three cams are installed on the camshaft actuated by one motor, and three sets of chewing trajectories can be generated simultaneously, one per oral chamber. Therefore, independent chewing tests using different parameter combinations can be applied to the chewing robot simultaneously. The RCC mechanisms consist of two plates and three elastic rods, which are uniformly distributed around the circumference and connected to both the plates. The occlusal force applied to each chewing chamber can be adjusted by adjusting the length of the pre‐tightened springs inside the rods of RCC mechanisms between the upper molar and the end of the follower. A passive food repositioning mechanism is also applied in each chamber; it has two inclined plates coated with Teflon, ensuring that saliva mixed food particles do not stick to the surfaces of the plates but would instead slide down onto the occlusal surfaces of the molar due to gravity after each cycle of occlusion. This mechanism achieves limited functionalities of the human tongue, and the absence of active tongue movements and sensory feedback is a significant limitation, particularly for accurately simulating intraoral bolus manipulation. All chambers are independently monitored through dedicated load cells, and the applied occlusal forces were calibrated before each test to ensure consistent force profiles across chambers. The artificial saliva is applied to the oral chamber via a precise peristaltic pump (LM60B, RZ02‐3‐1.6‐L; Hangzhou, China).

### 2.4 Design of Experiments

In this study, different chewing trajectories, occlusal forces, and saliva secretion rates were combined to simulate different food chewing behaviors and study the effects of the parameters on food texture changes during robotic chewing. Each factor had three levels. For chewing trajectories, Levels 1, 2, and 3 represent the measured data of chewing trajectories (Frisoli et al. 2017) with the identical chewing movements (15 mm) but varying lateral movements (0, 3.3, and 10.0 mm, respectively). For occlusal forces, experimental data (Jayakumar et al. 2023; Takaki et al. 2014) of children, adolescents, and adults with different occlusal forces of one side molar were used, with Levels 1, 2, and 3 representing 80, 150, and 350 N, respectively. For saliva secretion rates, clinical data of young and older adults (Affoo et al. 2015) were used, and Levels 1, 2, and 3 represent 1.0, 1.5, and 2.0 mL/min, respectively.

An orthogonal design of experiments (DoE), rather than the full factorial experiment, was used to improve efficiency (Freddi and Salmon 2019). In this study, L9.3.3 orthogonal table was applied, which indicates that a total of nine tests was required, with three factors, each having three levels, as shown in Table 2.

**TABLE 2:** The orthogonal design of experiments of robotic chewing experiments.

No.	Chewing trajectory	Occlusal force	Saliva rate
1	1	1	1
2	1	2	3
3	1	3	2
4	2	1	3
5	2	2	2
6	2	3	1
7	3	1	2
8	3	2	1
9	3	3	3

### 2.5 Measurement of Food Texture Variables

The size and weight of the peanuts and white bread applied to the robotic chewing tests are consistent with those chewed by human subjects in previous in vivo chewing experiments. For each group of tests, the food samples were chewed for 0%, 25%, 50%, 75%, and 100% of the number of chews, respectively, and the food textures of the samples were measured using a double compression test via the texture profile analyzer (CT3; Brookfield, MA, USA) after each chewing stage. The chewed food samples were placed in a lidded container, and the texture measurements were taken immediately to prevent the changes in food texture due to time and environmental factors. It should be noted that, although the size and weight of the food samples prepared for each chewing experiment were consistent, the size of the food samples after chewing is not uniform. To eliminate the impact of this inconsistency of the measurement results, a round probe with 20 mm in thickness and 50 mm in diameter, which is larger than the food samples, was used to ensure that the entire food samples were compressed evenly, to reduce the errors caused by the irregular shapes and sizes of the food samples. The heights of the food samples were measured and 80% of the value was set as the compression distance for each texture profile analysis (TPA) measurement, to eliminate the impact of varying sample heights after chewing on the measurements. The compression speed of the probe was 1.0 mm/s, and the trigger point load was 7 g. The hardness, adhesive force, adhesiveness, cohesiveness, springiness, springiness index, gumminess, chewiness, and chewiness index of food samples after each chewing stage were measured and recorded. The sensory and mathematical definitions of those variables were presented in the datasheet (Texture Pro operation instructions 2022), where the first six variables are directly measured, gumminess is calculated as: gumminess = hardness × cohesiveness, chewiness is calculated as chewiness = gumminess × springiness, chewiness index is a normalized version of chewiness used for cross‐sample comparisons. Real human chewing results were also measured and compared with robotic chewing results. Each test and measurement were repeated three times. Therefore, there was a total of 216 robotic chewing and 24 real human chewing tests measurements.

### 2.6 Statistical Analysis

The statistical analysis, principal component analysis (PCA) and partial least squares regression (PLSR) were carried out with MATLAB. The correlation analysis between the chewing behaviors of the robotic chewing and measured food texture variables over the chewing stages was presented by using PLSR, where the *x*‐variables were the chewing behaviors of the robot, and *y*‐variables were the food texture variables measured via TPA.

## 3 Results and Discussion

### 3.1 PCA Analysis of the Food Texture Variables

**FIGURE 2:**
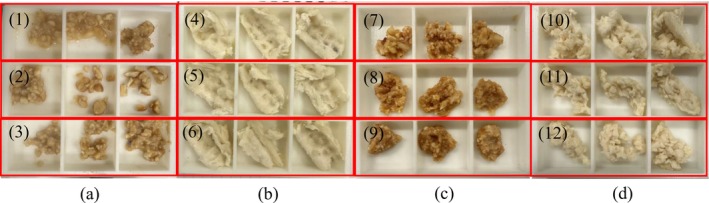
(a) In vitro chewing tests of peanuts: (1) test 3, (2) test 4, (3) test 8, (b) In vitro chewing tests of white bread: (4) test 2, (5) test 6 and (6) test 7, for 100% the chewing cycles required, (c) in vivo chewing tests of peanuts for (7) 25%, (8) 50%, (9) 100% and (d) in vivo chewing tests of white bread for (10) 25%, (11) 50%, (12) 100% the chewing cycles required.

The chewed samples of roasted peanuts and bread were collected in a box and then measured by a texture analyzer. Some representative chewed samples were shown in Figure 2. For roasted peanuts, the degree of food particle fragmentation and the extent of particle‐saliva mixing were relatively consistent across samples obtained under identical chewing behaviors; whereas noticeable variations were observed when different chewing behaviors were applied. In contrast, for white bread, the food samples remained relatively intact regardless of the chewing behaviors. This is attributed to the soft and elastic texture of bread, which makes it resistant to fragmentation through the compressive and shear forces generated by molar movements.

During robotic food chewing, the grinding and shearing of the molars and artificial saliva secretions to food samples led to a significant change in its texture variables over chewing cycles. Differences in the dominant texture variables perceived between roasted peanuts and white bread during the robotic chewing and their relationship with the chewing behaviors were found via the PCA. Four principal components (PCs) were found to represent the variables for both roasted peanuts and white bread. The loading coefficients describe the contribution of all original variables to all principal components. The values of the loading coefficients were shown in Table 3. For roasted peanuts, PC1 and PC2 mainly represent the food texture variables and chewing cycles while PC3 and PC4 mainly represent the chewing behaviors. The variance explained by PC1 to PC4 was 36.899%, 20.919%, 9.87%, and 8.985%, respectively, while the values of PC5 onwards have much smaller influence than that of first two PCs and were ignored (Greenacre et al. 2022). The cumulative variance explained rate was 76.674%, which indicates that the majority of the data information was explained by the principal components. For white bread, the physical meaning of its principal components was similar to that of roasted peanuts. The variance explained rate for PC1 to PC4 were 40.652%, 20.587%, 8.474%, and 8.296%, respectively, and the cumulative variance explained rate was 78.009%. This indicates that the amount of data variance explained by its principal components is greater than that explained by the principal components of peanuts.

**TABLE 3:** The loading coefficients values of PCA analysis for the PCA analysis of robotic chewing of roasted peanuts and white bread.

Food textures	Roasted peanuts			
	PC1	PC2	PC3	PC4
Chewing trajectory	−0.189	−0.394	−0.214	0.553
Occlusal force	−0.028	−0.377	0.128	−0.558
Saliva rate	0.067	0.194	0.698	0.37
Chewing cycles	0.829	−0.476	0.049	0.142
Hardness	−0.703	0.651	0.097	−0.069
Adhesive force	0.77	−0.362	0.293	−0.019
Adhesiveness	0.466	−0.118	0.347	−0.543
Cohesiveness	0.802	−0.396	0.139	0.193
Springiness	0.68	0.345	−0.404	−0.16
Springiness index	0.763	0.026	−0.438	0.039
Gumminess	0.429	0.689	0.356	0.132
Chewiness	0.62	0.7	−0.121	−0.057
Chewiness index	0.713	0.591	−0.033	0.096

Food textures	White bread			
	PC1	PC2	PC3	PC4
Chewing trajectory	−0.004	−0.072	0.603	−0.688
Occlusal force	0.074	0.185	0.659	0.284
Saliva rate	−0.013	−0.078	0.287	0.59
Chewing cycles	0.771	−0.243	−0.055	−0.181
Hardness	0.935	0.269	0.079	−0.004
Adhesive force	0.807	−0.092	−0.103	0.061
Adhesiveness	0.711	−0.284	−0.348	0.045
Cohesiveness	−0.713	0.537	−0.022	0.251
Springiness	−0.751	0.517	−0.095	−0.111
Springiness index	−0.541	0.559	−0.24	−0.225
Gumminess	0.831	0.479	0.095	0.088
Chewiness	0.476	0.858	−0.007	−0.037
Chewiness index	0.61	0.759	−0.058	−0.056

**FIGURE 3:**
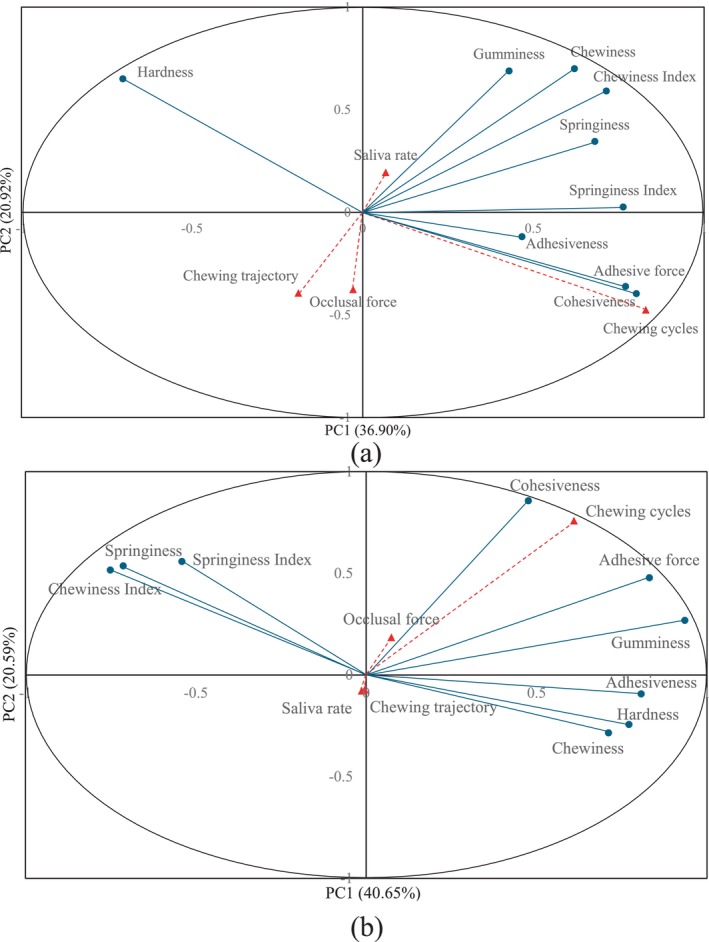
The correlation loading plot of PCA analysis of chewing behaviors and food texture variables of (a) roasted peanuts and (b) white bread samples during robotic chewing. The red triangles represent the chewing behavior variables and the blue circles represent the food texture variables measured by TPA.

The loading plots of the relation between all chewing behavior and food texture variables and the first two principal components of roasted peanuts and white bread were shown in Figure 3. As can be seen in Figure 3a, for robotic chewing of roasted peanuts, PC1 was mainly contributed by various food texture variables and the chewing cycles. Most of the food texture variables were located on the right half of the plot and far away from the origin, indicating that these variables had a large positive contribution to PC1. However, for roasted peanuts, the PCA loading plot showed that hardness had a large negative contribution to PC1, whereas cohesiveness, adhesive force, and springiness had strong positive contributions. Physiologically, this reflects the fragmentation process of hard foods: as mastication progresses, large particles are crushed (reduced hardness), while moisture absorption and deformation enhance adhesiveness and cohesiveness. This also suggests that cohesiveness and adhesiveness are not only physical responses but also serve as essential indicators of bolus formation. Therefore, it can be concluded that for robotic chewing of peanuts, the values of adhesive force and cohesiveness were highly correlated with the number of chewing cycles.

However, for the robotic chewing of white bread, the situation is different. As can be seen in Figure 3b, for robotic chewing of white bread, PC1 was also mainly contributed to by most of the food texture variables and chewing cycles. However, for white bread, PCA revealed that hardness increased in the initial chewing stages. This is attributed to the compressive deformation of soft bread, which leads to temporary compaction before saliva‐induced softening occurs. The positioning of springiness and springiness index on the opposite side of PC1 indicates that elasticity reduces as bolus water content rises. This suggests that PCA effectively captures the mechanical‐to‐rheological transformation process of bread, which is a hallmark of successful bolus preparation in elastic food types.

### 3.2 Correlation Analysis of Chewing Behaviors and Food Texture Variables

**FIGURE 4:**
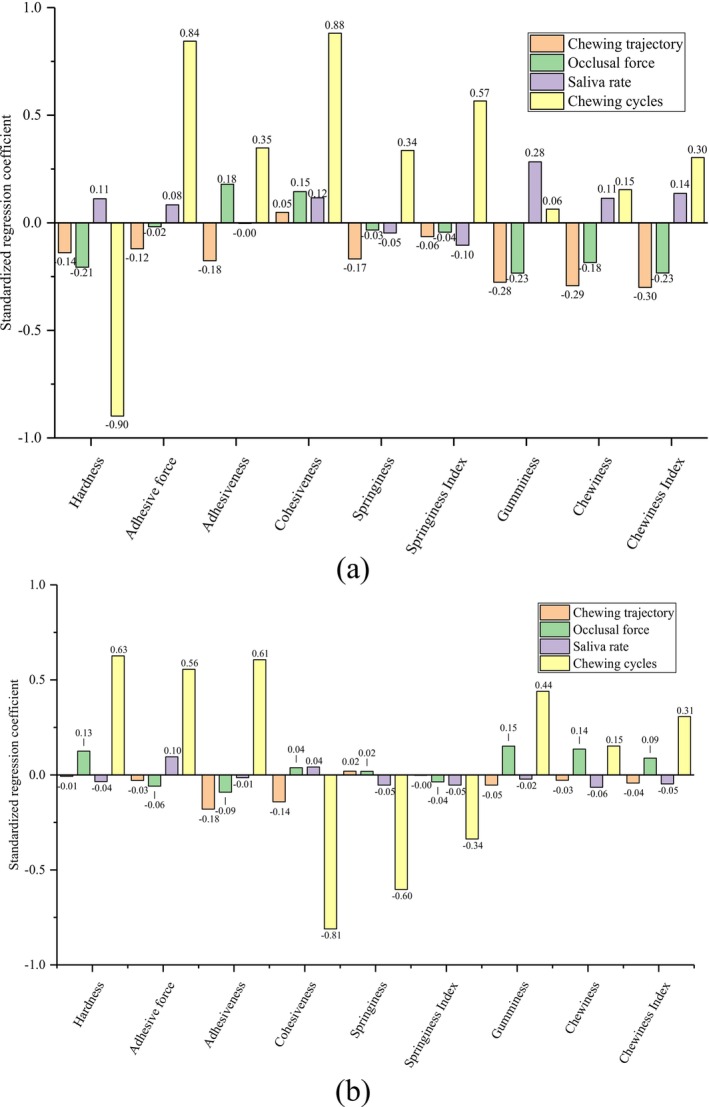
The standardized regression coefficients of PLSR analysis of chewing behaviors and food texture variables of (a) roasted peanuts and (b) white bread samples during robotic chewing.

The relationship between the chewing behaviors of robotic chewing and food texture variables measured by TPA was analyzed via PLSR. The results were shown in Figure 4. As can be seen in Figure 4a, for robotic chewing of roasted peanuts with various chewing behaviors, the regression coefficients of hardness, adhesive force, and cohesiveness had absolute values larger than 0.8, indicating that hardness in roasted peanuts was strongly negatively correlated with chewing cycles. This aligns with the expected mechanical breakdown of a hard food substrate, where molar forces disintegrate the structure into finer particles. Adhesive force and cohesiveness, however, were positively associated with chewing cycles. Physiologically, this reflects the dual role of mastication—not just reducing size but also facilitating the aggregation of particles into a swallowable bolus through saliva‐induced cohesion. Interestingly, gumminess and chewiness showed low regression coefficients, suggesting that these parameters are more sensitive to complex interactions, including PSD, saliva mixing dynamics, and occlusal trajectory. These nonlinear dependencies could explain why gumminess and chewiness, though perceptually important in oral texture evaluation, are not easily predicted by single behavioral inputs.

In the PLSR results for white bread in Figure 4b, cohesiveness exhibited a strong negative correlation with chewing cycles. This implies that, unlike harder foods, white bread's internal structure transitions from a cohesive to a fragmented state. Initially, bread has a spongy and interconnected matrix, but as mastication progresses, the porous network collapses and fragments, leading to decreased cohesiveness. This behavior is physiologically significant, as it suggests that bread transitions from an elastic structure to a more amorphous bolus as saliva is introduced and mixing occurs.

Meanwhile, hardness, adhesive force, and adhesiveness showed moderate positive correlations with chewing cycles. The increasing hardness during initial chewing may seem counterintuitive but can be explained by the compacting of the bread matrix under molar compression. As the water content from saliva is not immediately sufficient to soften the sample, the bread becomes temporarily denser. Later in the chewing process, the effect of saliva becomes dominant, gradually reducing structural resistance. This two‐phase behavior, which is the initial compaction followed by hydration, highlights the critical roles of occlusal force and saliva secretion rate in bread's textural evolution.

Springiness, on the other hand, had a moderate negative correlation with chewing cycles. This reflects the gradual loss of the bread's elastic recoil due to saturation with moisture and mechanical deformation. The structure's ability to rebound diminishes as the gluten network breaks down, indicating progression toward a bolus state. Notably, the weak correlations for gumminess, chewiness, and chewiness index suggest that these compound textural parameters are not linearly related to single mastication variables but may instead depend on more complex, time‐dependent interactions, such as microstructural collapse, saliva absorption dynamics, and deformation under compression.

### 3.3 Analysis of Food Texture Change Mechanisms

The overall effects of chewing behaviors on food texture variables were analyzed via PCA analysis and PLS regression. However, the mechanisms of food texture changes across chewing cycles, as well as the effects of different chewing behaviors across population groups on food texture variables, have not been thoroughly analyzed. From the previous analysis, it can be observed that the food texture variables can be divided into two categories. The first one is the texture variables that were highly correlated with the chewing cycles; the relationship between the two was close to linear. Another one is the texture variables that had low correlation coefficients with the chewing behaviors. These two categories were discussed separately. The TPA results of human subjects were also presented to compare the differences of in vivo and in vitro chewing.

**FIGURE 5:**
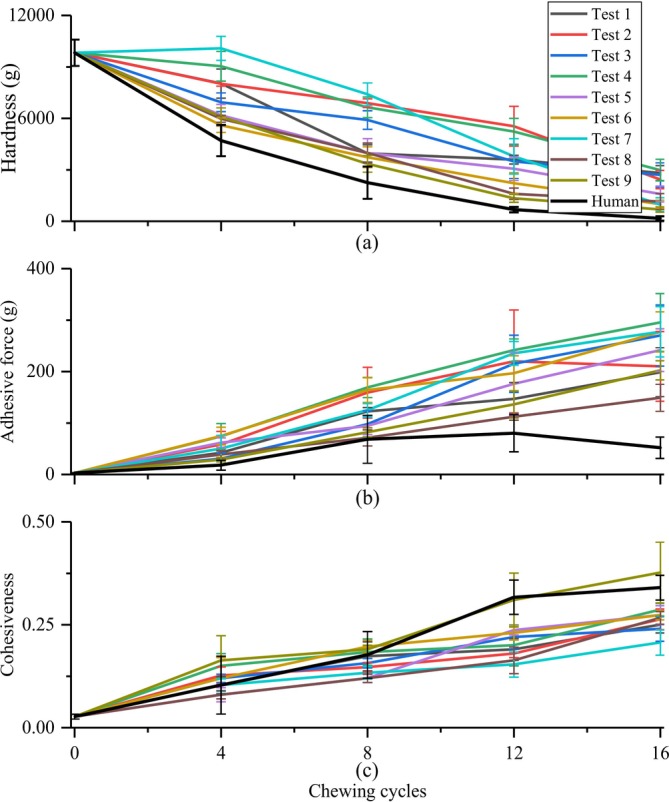
Changes in (a) hardness, (b) adhesive force, and (c) cohesiveness of roasted peanuts chewed by the robot under different chewing behaviors (Test 1–9, see Table 2 for parameter combinations) and by human subjects.

**FIGURE 6:**
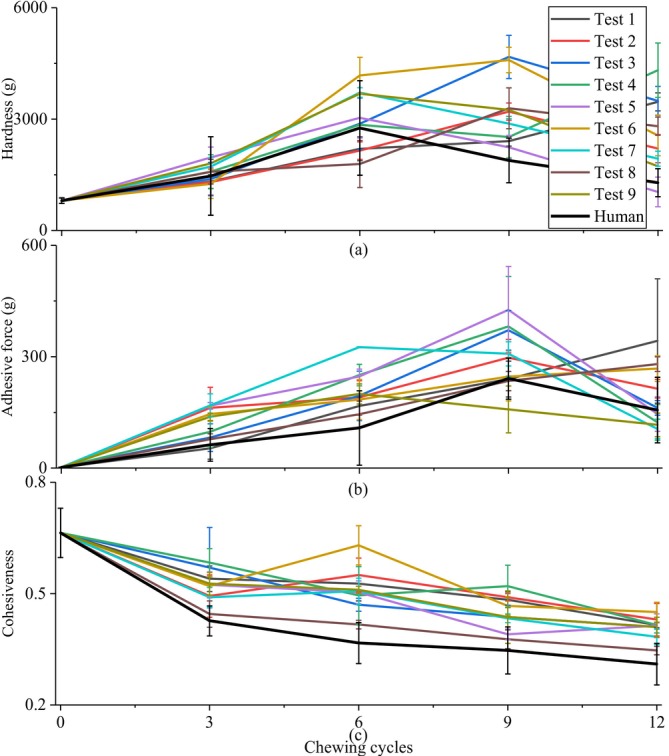
Changes in (a) hardness, (b) adhesive force, and (c) cohesiveness of white bread chewed by the robot under different chewing behaviors (Test 1–9, see Table 2 for parameter combinations) and by human subjects.

**FIGURE 7:**
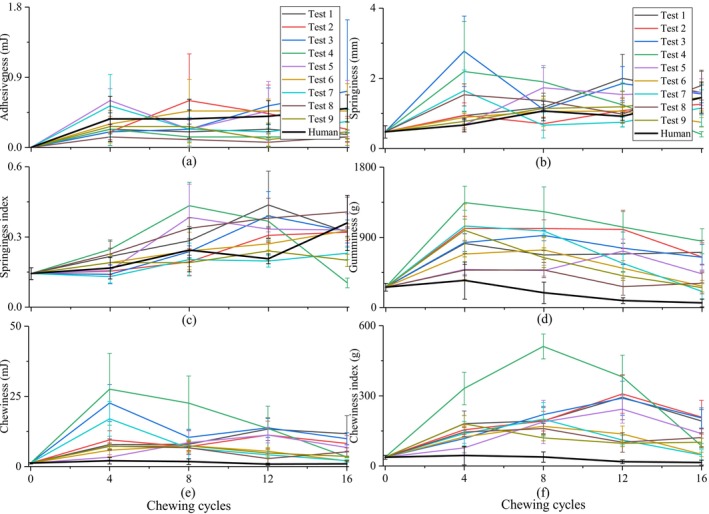
Changes in (a) adhesiveness, (b) springiness, (c) springiness index, (d) gumminess, (e) chewiness, and (f) chewiness index of the roasted peanut chewed by robot under different chewing behaviors (Test 1–9, see Table 2 for parameter combinations) and by human subjects.

**FIGURE 8:**
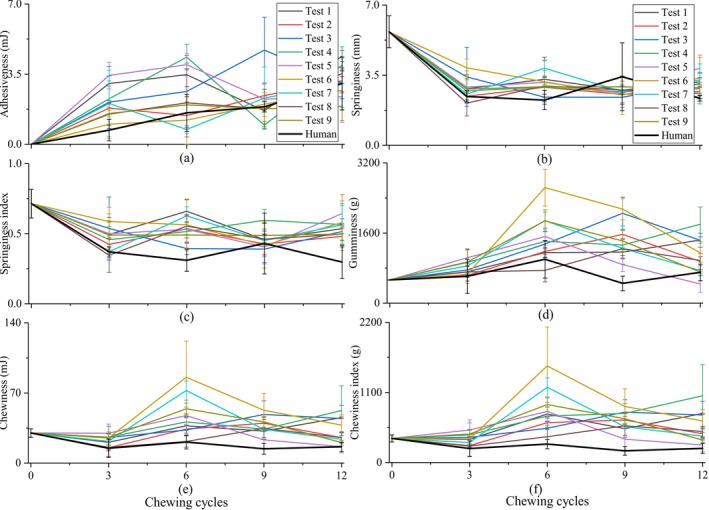
The changes of (a) adhesiveness, (b) springiness, (c) springiness index, (d) gumminess, (e) chewiness, and (f) chewiness index of the white bread chewed by robot under different chewing behaviors (Test 1–9, see Table 2 for parameter combinations) and by human subjects.

In Figures 5, 6, 7, 8, “Test 1–9” denoted the nine experimental conditions designed using an L9.3.3 orthogonal array, where three chewing behavior parameters were varied at three levels: chewing trajectory (0, 3.3, 10 mm lateral motion), occlusal force (80, 150, 350 N), and saliva secretion rate (1.0, 1.5, 2.0 mL/min), as shown in Table 2. Each test condition represents a unique combination of these three variables. For instance, Test 1 reflects minimal chewing effort (no lateral motion, low force, low saliva rate), whereas Test 9 represents the most intensive chewing condition (maximum trajectory, highest force, and highest saliva rate). This experimental design enables a structured analysis of how individual and combined chewing factors influence food texture evolution.

#### 3.3.1 Analysis of Food Texture Variables That Were Highly Correlated With Chewing Cycles

During robotic chewing of roasted peanuts, the evolution of hardness, adhesive force, and cohesiveness over successive chewing cycles, under various chewing behaviors for robotic chewing, as well as for human chewing, is presented in Figure 5. Overall, the trends in food texture changes observed during robotic chewing were largely consistent with those observed in real human chewing. Specifically, food hardness decreased progressively with an increasing number of chewing cycles, while adhesive force and cohesiveness increased over the same period.

As illustrated in Figure 5a, for roasted peanuts, hardness, adhesive force, and cohesiveness were primarily influenced by the chewing stages, with hardness decreasing by 81.5% on average over chewing cycles. Despite the general similarities, some discrepancies between robotic and human chewing results were evident, with variations in chewing behaviors influencing both the rate of textural change and the final texture of the food samples after chewing. For instance, the rate of hardness reduction was faster in human chewing compared to robotic chewing, and the final hardness of the food was lower. Among the robotic chewing tests, the hardness of the food samples in Tests 5, 6, 8, and 9, which correspond to conditions with either medium‐to‐high occlusal forces or increased lateral chewing trajectories, decreased more rapidly than others. These results suggest that an occlusal force exceeding 150 N, particularly when combined with lateral motion (Trajectory 2 or 3), enhances the molar shearing efficiency of the molars, accelerating the fragmentation of peanut particles. Humans can also better and actively place the food samples on molars due to tactile sensing and flexible manipulation enabled by tongues, resulting in more efficient chewing performance.

For adhesive force, depicted in Figure 5b, the results varied across different test conditions. Robotic chewing in Tests 2, 4, and 7 produced higher adhesive forces, whereas Tests 1, 8, and 9 showed lower values, indicating a general trend where higher saliva rates led to increased adhesive force. However, the results of Test 9 deviated from this pattern. In this case, the combination of high occlusal force and pronounced shearing action broke down peanut particles more effectively than in other tests, and the excessive addition of saliva fully mixed with the food particles, producing a more fluid‐like consistency rather than a semi‐solid, adhesive bolus. In contrast, human chewing results showed an initial increase followed by a decrease in adhesive force, consistently exhibiting lower values compared to robotic chewing. This can be attributed to the relatively low human saliva secretion rate at the early stage of mastication, leading to a slower increase in adhesive force. In the later stages, increased saliva secretion mixed with the food particles, eventually reducing the adhesive force as the bolus approached a swallowable consistency.

Regarding cohesiveness, as shown in Figure 5c, the trends observed for both human and robotic chewing were generally similar. Among the robotic chewing tests, Tests 6 and 9 exhibited higher cohesiveness, while Tests 7 and 8 demonstrated lower values. This pattern suggests that, in addition to the number of chewing cycles, occlusal force plays a dominant role in determining the cohesiveness of food samples. Higher occlusal forces facilitate more effective breakdown and mixing of food particles with saliva, forming a more uniform and cohesive bolus. This suggests that adolescents and adults with higher occlusal force can achieve more effective mastication of hard food than children with lower occlusal capabilities.

In this study, we observed that chewing behavior, particularly variations in chewing trajectory, occlusal force, and saliva flow rate, significantly influenced the transformation of food texture during mastication. As the number of chewing cycles increased, the food bolus gradually became softer and more cohesive, consistent with findings by (Wada et al. 2017), who reported that increased mastication resulted in reduced bolus hardness alongside increased adhesiveness and cohesiveness over time. Moreover, our results align with those of (Khramova et al. 2025), who demonstrated that voluntarily adjusting chewing behavior modulates the oral breakdown process and texture perception. They showed that faster chewing led to insufficient texture softening before swallowing, while slower chewing enhanced texture degradation and bolus cohesiveness, leading to higher acceptability of the food sample. These findings collectively underscore the critical role of mastication behavior in the dynamic evolution of food texture, validating the patterns observed in our robotic chewing experiments.

The textural changes of white bread during the chewing process were more complex than those observed in roasted peanuts. The evolution of these textural variables over successive chewing cycles, under various chewing behaviors for robotic chewing, as well as for human chewing, is illustrated in Figure 6. Overall, the trends in food texture changes between human and robotic chewing were largely consistent.

As shown in Figure 6a, the hardness of white bread increased initially and then decreased during both human and robotic chewing. The peak hardness was observed when the chewing cycles reached approximately 50% to 75% of the total number of cycles. This pattern can be attributed to the structural transformation of white bread: in the early stages of chewing, the compressive forces exerted by the molars caused the soft bread structure to become more compact, leading to a significant increase in hardness. As chewing progressed, the addition of saliva softened the bread and facilitated bolus formation; this two‐phase behavior indicates a transition from mechanical compaction to saliva‐driven softening. Thus, occlusal force primarily influenced the initial increase in bread hardness, while saliva played a dominant role in the subsequent decrease. These results indicate that adolescents and adults, who generally exhibit higher occlusal force and saliva secretion rates, are more effective at chewing soft and resilient foods compared to children.

For adhesive force, depicted in Figure 6b, different trends were observed across the chewing behaviors. In Tests 2, 3, 4, 7, and 9, the adhesive force initially increased and subsequently decreased; whereas in Tests 1, 6, and 8, it continuously increased throughout the chewing cycles. These results suggest that the saliva rate was the primary factor affecting changes in adhesive force. When the saliva rate was 1.0 mL/min, the adhesive force steadily increased due to the balanced addition of saliva. At higher saliva secretion rates of 1.5 and 2.0 mL/min, the adhesive force initially increased but later decreased as excessive saliva diluted the food samples, reducing their stickiness due to the increased water content. This trend was also observed in the results of human chewing.

Cohesiveness, as shown in Figure 6c, generally decreased with the increase of chewing cycles. Notably, the cohesiveness values in Tests 1, 2, and 3 were higher compared to those in Tests 7, 8, and 9, indicating that, in addition to the number of chewing cycles, the chewing trajectory was a key factor influencing cohesiveness. The pronounced shearing motion of the molars in Trajectory 3 tended to tear the food particles apart, leading to a significant reduction in cohesiveness. In contrast, the grinding motion in Trajectory 1 primarily compressed the food, preserving a firmer structure. This suggests that people who chew more laterally have higher chewing efficiency than those who chew vertically. The results from human chewing consistently showed lower cohesiveness values compared to robotic chewing. This is because the applied food repositioning mechanism in this manuscript can only simply place the food on the lower molar before each chewing, meaning it cannot reposition and mix the food as effectively as the human tongue during chewing to make it fully chewed and mix with saliva during chewing. Also, due to the compactness of the oral chamber, food of too large a size may not be fully broken down.

#### 3.3.2 Analysis of Food Texture Variables That Had Low Linear Correlation With the Chewing Behaviors

For the robotic chewing of roasted peanuts, the texture variables of adhesiveness, springiness, springiness index, gumminess, chewiness, and chewiness index exhibited low correlation with the chewing behaviors. The variations in these texture variables over the chewing cycles under different chewing behaviors during robotic chewing and human chewing are illustrated in Figure 7. For adhesiveness, springiness, and springiness index, the trends observed in human chewing closely aligned with those of robotic chewing, whereas the values of gumminess, chewiness, and chewiness index were consistently lower in human chewing compared to robotic chewing.

As shown in Figure 7a, adhesiveness values fluctuated with increasing chewing cycles rather than following the continuous increase observed in adhesive force. This discrepancy arises because adhesive force is a single‐point peak measurement reflecting the increasing stickiness of the food sample over the chewing cycles, whereas adhesiveness is a cumulative parameter influenced by the complexity of the separation process during measurements and is more susceptible to random experimental and measurement perturbations.

The results for springiness and springiness index, depicted in Figure 7b,c, respectively, reveal an initial increase followed by either stabilization or a slight decrease. Higher values were observed in Tests 1, 6, and 8, while lower values were found in Tests 4, 7, and 9, indicating that saliva rate was the dominant factor influencing the springiness index. Excessive saliva addition over the chewing cycles led to a reduction in the springiness of the food samples.

The trends of gumminess, chewiness, and chewiness index, presented in Figure 7d–f, were similar, characterized by an initial increase followed by a subsequent decrease. In robotic chewing, higher values were observed in Tests 4, 2, 3, and 1, while Tests 6, 8, and 9 exhibited lower values, suggesting that the combination of chewing trajectory and occlusal force played a key role in determining these texture variables. Specifically, the combination of a shearing‐effect trajectory and high occlusal force facilitated the breakdown of food samples and thorough mixing with saliva, leading to lower gumminess, chewiness, and chewiness index, which represent the energy required for disintegrating and preparing the samples for safe swallowing. This aligns with those of (Le Révérend et al. 2016), who demonstrated that mastication mechanics adapt dynamically even to subtle differences in texture, which in turn modifies the food's structural disintegration. This suggests that food breakdown and texture change are not solely determined by the initial texture of the food, but also by the neuromuscular strategy controlled chewing behaviors employed during chewing. This also suggests that adolescents and adults who exhibit greater lateral chewing trajectories demonstrate higher chewing efficiency. This also explains the consistently lower texture values in human chewing, as demonstrated in Figure 5a, where humans exhibited greater efficiency in crushing and softening hard foods.

Notably, the results from Test 7 showed a significant increase in these variables during the early stages of chewing, followed by a gradual decrease. This pattern can be attributed to the limited ability of the molar to fragment the food into smaller particles at the initial stages due to low occlusal force, despite the presence of a chewing trajectory with a pronounced shearing effect. As chewing cycles progressed, continuous saliva addition softened the food samples, which were subsequently broken down into smaller particles and thoroughly mixed with saliva, ultimately resulting in lower values for gumminess, chewiness, and chewiness index in the final stages of chewing.

For the robotic chewing of white bread, adhesiveness, springiness, springiness index, gumminess, chewiness, and chewiness index exhibited low linear correlations with the chewing behaviors. The variations in these textural variables over the chewing cycles, under different chewing behaviors during robotic and human chewing, are presented in Figure 8. Consistent with the findings for roasted peanuts, the results of adhesiveness, springiness, and springiness index for human and robotic chewing were generally similar, whereas the values for gumminess, chewiness, and chewiness index were lower in human chewing compared to robotic chewing.

As shown in Figure 8a, the values of adhesiveness fluctuated over the chewing cycles, similar to the trends observed for roasted peanuts in Figure 7a. This fluctuation can be attributed to the complexity of the separation process during measurements, which is sensitive to experimental and measurement conditions. The results for springiness and springiness index over the chewing cycles, depicted in Figure 8b and Figure 8c, respectively, demonstrated an initial decrease during the first 25% of the total chewing cycles, followed by slight fluctuations. This reflects the loss of the bread's elastic recoil due to mechanical deformation during the initial chewing stages. The structure's ability to rebound diminishes as the gluten network breaks down, indicating progression toward a bolus state.

The results for gumminess, chewiness, and chewiness index, shown in Figure 8d–f, displayed similar trends and final values. However, during the mid‐stage of chewing, where the chewing cycles reached approximately 50%, the results for Tests 3, 6, 7, and 9 were higher compared to other tests. This suggests that occlusal force was the dominant factor influencing these textural variables. As previously discussed, an increase in occlusal force leads to greater hardness in white bread during the middle stage of chewing. Consequently, harder foods require more energy for proper disintegration and preparation for swallowing, resulting in higher values for gumminess, chewiness, and chewiness index. In the later stages of chewing, the continuous addition of saliva softened the food samples, leading to a decrease in these textural values, indicating a higher chewing efficiency of adults than children when chewing resilient foods. This is also proved by (Iguchi et al. 2015), who confirmed that distinct chewing behaviors result in different trajectories of physical texture change, such as changes in hardness and elasticity, reinforcing the notion that mastication is a texture‐modulating process in itself.

In human mastication, lateral movements of the mandible play a crucial role in achieving efficient food comminution and bolus formation. Studies have reported that typical lateral excursions during chewing range between 5.5 and 9.5 mm, depending on food hardness, dry matter content, and density (Le Révérend et al. 2016). In our robotic chewing system, the lateral motions were preset to 0, 3.3, and 10 mm, covering a comparable range. However, unlike human chewing, where lateral amplitude and frequency dynamically adapt to sensory feedback, our cam‐based mechanism imposes fixed motion trajectories throughout the chewing cycles. This lack of adaptability may influence the efficiency of particle breakdown and mixing. For example, while the 10 mm setting simulates an exaggerated lateral sweep, it does not modulate in response to bolus texture changes. Therefore, although our robot captures the range of human lateral motions, it cannot replicate the adaptive modulation observed in vivo. This fundamental distinction should be considered when interpreting the comminution patterns and bolus properties generated by the robotic system.

Based on the above discussion, the changes of food texture during the masticatory process of different groups of people can be summarized. In general, adolescents and adults with higher occlusal force and saliva secretion rate have a higher chewing performance than children. Also, it should be noted that with the level of the same occlusal force and saliva secretion rate, people with a chewing trajectory with large lateral movements have a higher chewing efficiency than others.

## 4 Conclusions

This study investigated the changes in food texture of roasted peanuts and white bread under different robotic chewing behaviors. Results indicated that for roasted peanuts, hardness, adhesive force, and cohesiveness exhibited strong correlations with chewing cycles, suggesting a close linear relationship between these variables and the number of chews. In contrast, for white bread, the correlations of these variables with chewing cycles were less pronounced. Other texture variables for both roasted peanuts and white bread showed low correlation coefficients, implying nonlinear relationships between these variables and the chewing behaviors.

The mechanisms underlying food texture changes during robotic chewing were analyzed and compared with those observed in human chewing. The masticatory performance within different groups of people was also analyzed. For roasted peanuts, the changes in hardness, adhesive force, and cohesiveness were primarily influenced by the chewing stages, whereas springiness and springiness index were mainly dependent on saliva secretion rate. The variations in gumminess, chewiness, and chewiness index were closely associated with the combined effects of chewing trajectory and occlusal force. For white bread, hardness was predominantly determined by occlusal force during the early stage of chewing and by saliva secretion rate in the later stage. Adhesive force was mainly influenced by saliva secretion rate, while cohesiveness was largely affected by chewing trajectory. Unlike roasted peanuts, white bread's springiness and springiness index showed no significant dependence on chewing behaviors. The gumminess, chewiness, and chewiness index of white bread were primarily affected by saliva secretion rate throughout the chewing process.

However, there are still some limitations in this study. For example, only two healthy male participants were included, which limits generalizability for in vivo and in vitro comparisons and restricts their broader applicability. Future work should include larger and more diverse populations. Biotene mouthwash was used as an artificial saliva substitute in this study. However, it lacks mucin proteins, which potentially affects the relevance of bolus cohesion and lubrication outcomes; therefore, collected human saliva or synthetic formulations with α‐amylase or mucin should be applied in future studies to better model physiological conditions. While the passive food repositioning mechanism provides limited functionalities of food particles repositioning, it does not fully replicate the complicated biological repositioning and shaping performed by the tongue in vivo. This limitation likely affects the realism of bolus formation and should be seriously addressed in the future.

In conclusion, this study systematically examined the texture changes of roasted peanuts and white bread during robotic chewing, analyzed the correlations between chewing behaviors and food texture variables, and elucidated the mechanisms underlying these texture changes. Our findings demonstrate the potential of robotic chewing as a tool for mimicking and evaluating mastication under controlled in vitro environments, offering valuable insights into the dynamics of oral food processing.

## Author Contributions

**Xudong Wang:** investigation, methodology, formal analysis, writing – original draft. **Bangxiang Chen:** methodology, writing – review and editing. **Jaspreet Dhupia:** investigation, writing – review and editing, supervision. **Macro Morgenstern:** methodology, writing – review and editing. **Weiliang Xu:** methodology, supervision, project administration, writing – review and editing.

## Ethics Statement

This study was approved by the Human Participants Ethics Committee of the University of Auckland (Reference number: UAHPEC26909).

## Consent

Writing informed consent was obtained from all study participants.

## Conflicts of Interest

The authors declare no conflicts of interest.

## Data Availability

The data that support the findings of this study are available on request from the corresponding author. The data are not publicly available due to privacy or ethical restrictions.
